# Monthly Prices of Substances Used in Pharmacy

**Published:** 1814-09

**Authors:** 


### *Monthly Prices of* SUBSTANCES *used in* PHARMACY,

At Messrs. SELWAY and HENLEY's (Chemical and Pharmaceutical Laboratory,) 35, Upper Mary-le-bone Street, Portland Place.

| | s. | d. |
|---|---|---|
| Acetum Scillæ lb. | 2s. | 0d. |
| Abietis Resina | 2 | 3 |
| Acaciæ Gummi | 2 | 3 |
| —— elect. | 3 | 3 |
| —— Pulvis | 3 | 8 |
| Acidum Aceticum cong. | 3 | 6 |
| —— Benzoicum oz. | 6 | 0 |
| —— Boracicum | 2 | 0 |
| —— Oxalicum lb. | 20 | 0 |
| —— Nitricum | 4 | 6 |
| —— Nitrosum | 2 | 6 |
| —— Muriaticum | 0 | 8 |
| —— Sulphuricum | 0 | 6 |
| —— Arom. | 5 | 0 |
| Alcohol sp. gr. 815 M. lb. | 5 | 0 |
| Æther sulphur. sp. gr. 789 | 10 | 0 |
| —— rectificatus sp. gr. 744 | 12 | 0 |
| Ærugo lb. | 7 | 0 |
| Aloes spicatæ extractum | 5 | 6 |
| —— vulgaris extractum | 4 | 6 |
| —— Barbadoes | 5 | 0 |
| Alumen | 0 | 4 |
| —— exsiccatum | 0 | 8 |
| Ammoniæ Murias Ang. | 2 | 9 |
| —— Exot. | 2 | 2 |
| —— Carbonas | 3 | 6 |
| —— Nitras | 5 | 6 |
| Amygdalæ Amaræ | 1 | 6 |
| —— Dulces | 5 | 8 |
| Ammoniacum (Gutt.) | 6 | 6 |
| —— (Commun.) | 3 | 6 |
| Anthemidis Flores (com.) | 1 | 9 |
| —— (opt.) | 2 | 0 |
| Antimonium | 3 | 0 |
| Antimonii Sulphuretum | 1 | 0 |
| —— Aurum. Sulph. | 7 | 0 |
| —— Oxydum | | |
| —— Murias | 3 | 9 |
| Antimonium Tartarizatum | 5 | 0 |
| Aqua Anethi cong. | 3 | 0 |
| —— Carui | 3 | 0 |
| —— Cinnamomi vera | 18 | 0 |
| —— Menthæ piperitæ | 2 | 6 |
| —— pulegii | 2 | 6 |
| —— Sativæ | 2 | 6 |
| —— Pimentæ | 2 | 9 |
| —— Rosæ | 5 | 0 |
| —— Cassiæ | 3 | 6 |
| Arsenici Oxydum lb. | 1 | 0 |
| Argenti Nitras | 6 | 9 |
| Assafœtida Gummi-resina | 6 | 6 |
| Aurantii Cortex | 2 | 6 |
| Balsamum Peruvianum | 33 | 0 |
| —— Tolutanum | 15 | 0 |
| Barytæ Murias | 6 | 0 |
| Benzoinum elect. | 11 | 0 |
| Bismuthum | 3 | 0 |
| —— Oxydum | 16 | 0 |
| —— Nitras | 16 | 0 |
| Calamina præp. | 0 | 3 |
| Calumbæ Radix | 2 | 6 |
| Calx | 1 | [illegible] |

| | s. | d. |
|---|---|---|
| Cambogia opt. | 9s. | 6d. |
| Camphora | 8 | 6 |
| Canella Cortex | 3 | 4 |
| Cardamomi Semina opt. | 12 | 0 |
| Cascarillæ Cortex | 5 | 6 |
| Castoreum unc. | 4 | 0 |
| Caryophilli lb. | 11 | 0 |
| Cassia Cortex | 8 | 0 |
| Catechu Extractum | 2 | 3 |
| Cetaceum | 3 | 0 |
| Cera flava | 3 | 6 |
| —— alba | 3 | 0 |
| Ceratum Cetacei | 3 | 0 |
| —— Resinæ | 2 | 8 |
| —— Calamina | 3 | 0 |
| —— Plumbi superacet. | 4 | 0 |
| —— Lyttæ | 4 | 0 |
| —— Sabinæ | 2 | 8 |
| —— Plumbi Carb. | 3 | 0 |
| —— Comp. | 3 | 8 |
| —— Saponis | 4 | 6 |
| Cinchonæ cordifoliæ Cortex (yellow) | 9 | 0 |
| —— lancifoliæ Cortex (quilled) | 13 | 0 |
| —— oblongifoliæ Cortex (red) | 16 | 0 |
| Cinnamomi Cortex | 17 | 0 |
| Coccus (Coccinella) | 66 | 0 |
| Colocynthidis | 7 | 6 |
| Copaiba | 7 | 0 |
| Colchici Radix | 3 | 7 |
| Cornu usti | 1 | 0 |
| Cretæ ppt. | 0 | 8 |
| Croci stigmata | 60 | 0 |
| Cupri sulphas | 1 | 0 |
| Cuprum ammoniatum | 6 | 0 |
| Curcuma Pulvis | 2 | 6 |
| Cuspariæ Cortex | 4 | 0 |
| Confectio Aromatica | 8 | 6 |
| —— Aurantii | 2 | 9 |
| —— Opii | 4 | 6 |
| —— Rosæ Gallicæ | 1 | 10 |
| —— Caninæ | 2 | 0 |
| —— Scammoniæ | 15 | 0 |
| —— Sennæ | 2 | 0 |
| —— Amygdalæ | 5 | 0 |
| Emplastrum Ammoniaci | 8 | 0 |
| —— Cumini | 2 | 0 |
| —— Galbani Comp. | 2 | 6 |
| —— Lyttæ | 6 | 6 |
| —— Opii | 2 | 6 |
| —— Plumbi | 1 | 6 |
| —— Resinæ | 1 | 6 |
| —— Roborans | 2 | 0 |
| —— Saponis | 2 | 6 |
| —— Hydrargyri | 2 | 6 |
| —— cum Amm. | 6 | 0 |
| Extractum Anthemidis unc. | 1 | 0 |
| —— Belladonnæ | 1 | 0 |
| —— Cinchonæ | 3 | 0 |
| —— resinos. | 4 | 0 |

| | | |
|---|---|---|
| Extractum Colocynthidis | 2s. | 0d. |
| —— comp. | 1 | 0 |
| —— Conii | 0 | 6 |
| —— Gentianæ | 0 | 5 |
| —— Glycyrrhizæ .. lb. | 3 | 0 |
| —— Cascarillæ ... unc. | 3 | 0 |
| —— Hæmatoxyli | 0 | 5 |
| —— Humuli | 0 | 9 |
| —— Hyoscyami | 1 | 0 |
| —— Jalapæ 1*s.* 6*d.* Res. | 2 | 8 |
| —— Opii aquos | 3 | 8 |
| —— resinos | 3 | 0 |
| —— Papaveris | 0 | 6 |
| —— Rhæi | 2 | 4 |
| —— Sarsaparillæ | 0 | 10 |
| —— Taraxaci | 0 | 6 |
| Ferri carbonas lb. | 1 | 2 |
| —— sulphas | 1 | 0 |
| Ferrum Ammoniatum | 3 | 6 |
| —— Tartarizatum | 4 | 0 |
| Galbani Gummi-resinæ | 8 | 6 |
| Gentianæ Radix | 1 | 3 |
| Guaiaci Resina | 8 | 0 |
| Hydrargyrus purificatus | 5 | 6 |
| —— cm Creta | 3 | 6 |
| Hydrargyri Oxymurias | 8 | 0 |
| —— Submurias | 8 | 6 |
| —— Hydros. | 10 | 0 |
| —— Nitrico-Oxydum .. | 8 | 6 |
| —— Oxydum Cinereum | 16 | 0 |
| —— Rubrum | 5 | 0 |
| —— Sulphuretum Nig. | 3 | 6 |
| —— Rub. | 8 | 0 |
| —— Præcipitatus Alb. | 8 | 0 |
| Hellebori nigri Radix | 2 | 6 |
| Ipecacuanhæ Radix | 20 | 0 |
| —— Pulvis | 21 | 0 |
| —— Compos. Pulv. lb. | 8 | 0 |
| Jalapæ Radix | 6 | 0 |
| —— Pulvis | 6 | 6 |
| Kino | 12 | 0 |
| Liquor Plumbi Acetatis M. lb. | 1 | 0 |
| —— Ammoniæ .... P.L. | 2 | 8 |
| —— Arsenicalis | 3 | 0 |
| —— Potassæ | 0 | 9 |
| —— Vol. Corn. Cerv. | 1 | 0 |
| Linimentum Camphoræ comp. | 5 | 6 |
| —— Saponis comp. | 5 | 6 |
| Lichen Islandicus ... P. lb. | 1 | 9 |
| Lini Semina | 0 | 6 |
| —— Pulvis | 0 | 4 |
| Lyttæ | 10 | 0 |
| Magnesia | 8 | 6 |
| —— Carbonas | 3 | 0 |
| —— Sulphas | 1 | 2 |
| Manna optima | 6 | 6 |
| —— communis | 5 | 0 |
| Mel Rosæ | 2 | 6 |
| —— Despumat | 3 | 0 |
| Nuces Moschatæ | 25 | 0 |
| Moschus pod, 24*s.* in gr. unc | 40 | 0 |
| Mastiche lb. | 5 | 6 |
| Myristicæ Nuclei | 25 | 0 |
| Myrrha elect. | 6 | 6 |
| Olibanum | 3 | 0 |
| Opoponax | 23 | 0 |

| | | |
|---|---|---|
| Opium (Turkey) | 34s. | 0d. |
| Opium (East India) lb. | 32 | 0 |
| Oleum Amygdale lb. | 3 | 8 |
| —— Anisi unc. | 2 | 6 |
| —— Anthemidis | 5 | 0 |
| —— Cinnamomi Cassiæ | 8 | 6 |
| —— Caryophili | 5 | 0 |
| —— Cajuputi | 8 | 0 |
| —— Carui | 1 | 3 |
| —— Juniperi | 0 | 9 |
| —— Lavandulæ | 4 | 0 |
| —— Lini cong. | 8 | 6 |
| —— Menthæ piperitæ unc. | 4 | 6 |
| —— viridis | 3 | 0 |
| —— Olivæ opt. cong. | 24 | 0 |
| —— secundum | 16 | 0 |
| —— Origani unc. | 2 | 0 |
| —— Pimentæ | 4 | 6 |
| —— Ricini optim. (per bottle) | 10 | 0 |
| —— secund. | 7 | 0 |
| —— Rosmarini unc. | 0 | 9 |
| —— Succini .. 2*s.* 4*d.* rect. | 2 | 6 |
| —— Sulphuretum lb. | 1 | 6 |
| —— Terebinthinæ rectif. .. | 2 | 0 |
| Oxymel Scillæ | 3 | 0 |
| Papaveris Capsulæ (per 100) | 2 | 4 |
| Plumbi Carbonas lb. | 0 | 8 |
| —— Superacetas | 2 | 2 |
| —— Oxydum semi-vitreum | 0 | 6 |
| Potassa Fusa unc. | 0 | 6 |
| —— cum Calce | 1 | 6 |
| Potassæ Nitras lb. | 1 | 0 |
| —— Acetas | 8 | 0 |
| —— Subcarbonas | 1 | 2 |
| —— Carbonas | 4 | 0 |
| —— Sulphas | 1 | 0 |
| —— Sulphuretum | 1 | 6 |
| —— Supersulphas | 3 | 0 |
| —— Tartras | 3 | 0 |
| —— Supertartras | 1 | 9 |
| Pilulæ Hydrargyri | 4 | 6 |
| —— Aloes comp. | 8 | 0 |
| —— cum Colocynth. | 16 | 0 |
| —— Saponis cum Opio .. | 16 | 0 |
| —— Scillæ Compos. | 6 | 6 |
| —— e Styrace | 32 | 0 |
| Piper longum | 2 | 6 |
| —— Nigrum lb. | 2 | 6 |
| Pimento lb. | 2 | 6 |
| Pulvis Aloes cum Canella | 6 | 6 |
| —— Compositus | 10 | 0 |
| —— Antimonialis | 7 | 0 |
| —— Cinnamomi Comp .. | 16 | 0 |
| —— Cornu cervi cum Opio | 16 | 0 |
| —— Cretæ Composit. | 8 | 0 |
| —— cum Opio | 9 | 0 |
| —— Kino Compositus | 12 | 0 |
| —— Scammoniæ Comp. unc. | 2 | 6 |
| —— Sennæ Composit. .. lb. | 8 | 0 |
| —— Tragacanthæ Comp. .. | 5 | 0 |
| Resina Flava | 0 | 5 |
| —— Nigra | 0 | 5 |
| Rosæ Folia | 9 | 0 |
| Rad. Quassiæ | 1 | 6 |
| Rhæi Radix (Russia) | 30 | 0 |
| —— (East India) | 13 | 6 |

| | | |
|---|---|---|
| Rhæi Radix Pulvis | 14s. | 6d. |
| Sapo (Spanish) | 2 | 6 |
| Sarsaparilla Radix Inc. | 5 | 0 |
| Scammoniæ Gum-Resina unc. | 2 | 6 |
| Scilla recens | 1 | 3 |
| ——— siccata | 5 | 0 |
| ——— ——— pulvis | 6 | 0 |
| Senegæ Radix | | |
| Sennæ Fo ia Opt. Alexand. | 6 | 6 |
| Serpentariæ Radix | 30 | 0 |
| Simaroubæ Cortex | 4 | 0 |
| Sodæ Boras | 4 | 6 |
| ——— Carbonas | 7 | 0 |
| ——— Subcarbonas | 1 | 4 |
| ——— ——— exsiccata | 2 | 8 |
| Sinapis super. | 2 | 6 |
| ——— secund. | 2 | 0 |
| Sodæ Sulphas | 0 | 8 |
| ——— Phosphas | 2 | 8 |
| ——— Acetas unc. | 0 | 10 |
| Soda tartarizata lb. | 2 | 4 |
| Spongia usta unc. | 1 | 3 |
| Spiritus Ammoniæ M. lb. | 3 | 6 |
| ——— ——— aromaticus | 4 | 6 |
| ——— ——— fœtidus | 3 | 6 |
| ——— ——— succinatus | 4 | 6 |
| ——— Cinnamomi ver. | 3 | 6 |
| ——— Juniperi Compos. | 3 | 0 |
| ——— Lavandulæ | 4 | 6 |
| ——— ——— Compos. | 5 | 0 |
| ——— Myristicæ | 3 | 0 |
| ——— Pimentæ | 3 | 0 |
| ——— Rosmarini | 3 | 6 |
| ——— Ætheris Aromaticus | 6 | 0 |
| ——— ——— Compositus | 6 | 0 |
| ——— ——— Nitrici sp. gr. 860 | 5 | 6 |
| ——— ——— Sulphurici | 6 | 0 |
| ——— Vini rectificatus | 26 | 0 |
| Syrupus Aurantii | 1 | 8 |
| ——— Rutæ | 1 | 8 |
| ——— Papaveris | 2 | 0 |
| ——— Violæ | 2 | 10 |
| ——— Rhei | 2 | 10 |
| ——— Rhamni | 2 | 0 |
| ——— Croci | 3 | 0 |
| ——— Tolutanus | 2 | 0 |
| ——— Rheados | 2 | 0 |
| ——— Mori | 2 | 6 |
| Sulphur | 0 | 6 |
| ——— Lotum | 1 | 0 |
| ——— Præcipitatum | 1 | 0 |
| Tamarindus opt. | 2 | 6 |
| Terebinthina Vulgaris | 0 | 9 |
| ——— Canadensis | 8 | 0 |
| ——— Chia | 6 | 0 |
| Tragacantha Gummi | 10 | 0 |
| ——— Pulvis | 10 | 6 |
| Tinctura Aurantii M. lb. | 3 | 9 |
| ——— Aloes | 4 | 0 |
| ——— ——— Composita | 9 | 6 |
| ——— Assafœtidæ | 5 | 6 |
| ——— Balsa. Tolut. | 5 | 6 |
| ——— Benzoini Comp. | 6 | 9 |
| ——— Camphoræ Comp. | 4 | 0 |

| | | |
|---|---|---|
| Tinctura Cardamomi | 4s. | 0d. |
| ——— ——— Comp. | 4 | 0 |
| ——— Calumbæ | 3 | 9 |
| ——— Capsici | 3 | 9 |
| ——— Cascarillæ | 4 | 6 |
| ——— Castorei | 7 | 0 |
| ——— Catechu | 4 | 0 |
| ——— Cinchonæ | 6 | 0 |
| ——— ——— Compos. | 6 | 0 |
| ——— Cinchonæ Ammon. | 7 | 0 |
| ——— Croci | 6 | 6 |
| ——— Digitalis | 4 | 6 |
| ——— Ferri Ammoniati | 4 | 8 |
| ——— Cinnamomi | 4 | 6 |
| ——— ——— Comp. | 4 | 6 |
| ——— Gentianæ Comp. | 4 | 0 |
| ——— Guaiaci | 6 | 0 |
| ——— ——— Ammon. | 6 | 0 |
| ——— Hellebori Nigri | 4 | 6 |
| ——— Humuli | 5 | 0 |
| ——— Hyoscyami Nig. | 4 | 6 |
| ——— Jalapæ | 4 | 6 |
| ——— Japonica | 4 | 6 |
| ——— Ferri Muriatis | 4 | 8 |
| ——— Kino | 5 | 0 |
| ——— Lyttæ | 3 | 9 |
| ——— Myrrhæ | 4 | 6 |
| ——— Opii | 7 | 0 |
| ——— ——— Camphorat. | 4 | 6 |
| ——— Quassiæ | 3 | 9 |
| ——— Rhei | 4 | 6 |
| ——— ——— Comp. | 4 | 0 |
| ——— Scillæ | 4 | 6 |
| ——— Sennæ | 4 | 9 |
| ——— Serpentariæ | 6 | 0 |
| ——— Valerianæ | 5 | 6 |
| ——— ——— Amm. | 4 | 6 |
| ——— Zingiberis | 5 | 6 |
| Valerianæ Radix | 1 | 8 |
| Veratri Radix | 1 | 6 |
| Unguentum Hydrargyri fort | 5 | 0 |
| ——— ——— Mit. | 3 | 0 |
| ——— ——— Nitratis | 3 | 3 |
| ——— ——— Nitrico-oxy. | 2 | 9 |
| ——— Sulphuris Comp. | 1 | 9 |
| ——— Sambuci | 2 | 0 |
| ——— ——— Virid | 1 | 8 |
| ——— Altheæ | 2 | 0 |
| ——— Cetacei | 3 | 6 |
| ——— Veratri | 1 | 8 |
| ——— Zinci | 2 | 0 |
| ——— Hyd. præcip. Alb. | 3 | 0 |
| ——— Picis | 1 | 6 |
| Uvæ Ursi Folia | 2 | 4 |
| Vinum Aloes | 3 | 6 |
| ——— Antimoniale | 3 | 0 |
| ——— Ferri | 3 | 6 |
| ——— Ipecacuanhæ | 5 | 6 |
| ——— Opii | 8 | 0 |
| Zinci Oxydum | 4 | 6 |
| ——— Sulphas purif. | 2 | 0 |
| ——— Acetas unc. | 1 | 6 |
| Zincum lb. | 1 | 6 |
| Zingiberis Radix opt. | 2 | 4 |

French Leeches, equal to English, 25s. per hundred, or 3s. 6d. per dozen.—English ditto, true, 12s. per dozen.—Essential Salt of Lemons, 4s. 6d. per dozen.

